# Atlanta Medical College

**Published:** 1871

**Authors:** 


### *Atlanta Medical College.*

The pleasant associations and labors of the present course of lectures in this institution are now about coming to a close. The anxieties, too, on the part of student and teacher, incident to the closing scenes of a collegiate education, will soon have an end. Those who are so soon to terminate their pupilage in order to assume the duties of professional life, look forward in the future to responsibilities still weightier, and to perplexities more embarrassing than any met with heretofore.

The season of 1871, which commenced on the first day of May, will close on the first day of September, with the usual commencement exercises. On that day, also, will be inaugurated an Annual Re-union of the Graduates of this College, to which we look forward with pleasure, as a means of bringing together every year those for whom we formed a warm attachment, and in whom we feel an abiding interest. We hope the contemplated meeting of graduates to form an Alumni Association, will be largely attended, and a satisfactory organization effected. Such associations work much good, not only to those connected with them, but to the various communities in which the members live. In the Annual meetings, interchange of views, and scientific discussions, should form a part of the proceedings.

The present course of Lectures was commenced under the most embarrassing circumstances, but has been more successful, in many respects, than usual. The instruction, both theoretical and practical, has never been exceeded at any previous session, and the number of applicants for graduation more than a fourth, and the entire class nearly one-third larger than that of last year.

The vacancies in the Faculty, occurring at the beginning of the session, by the death of Dr. Jones and by other causes, affected to some extent, the prospects of success. In addition to this, the pecuniary embarrassment which pervades the whole country, to an extent unprecedented since the organization of the institution, greatly diminished the number of the present class. Many who had, during the first of the year, made known their determination to attend, were prevented from doing so by the great scarcity of money.

We have reason to hope for a favorable change in the financial condition of the country by the beginning of another year, and with this we have the prospect of a largely increased class for the session of 1872.

